# Prognostic Value of Multimodal Cardiac Magnetic Resonance Parameters in Patients with Nondilated Left Ventricular Cardiomyopathy and Reduced Left Ventricular Ejection Fraction

**DOI:** 10.3390/jcm15030918

**Published:** 2026-01-23

**Authors:** Chunlong Yan, Shuang Li, Baiyan Zhuang, Shujuan Yang, Jiayi Liu, Jiangjun Qin, Lei Xu

**Affiliations:** 1 Department of Radiology, Beijing Anzhen Hospital, Capital Medical University, Beijing Institute of Heart, Lung, and Blood Vessel Diseases, Beijing 100029, China; yclky2012@126.com (C.Y.);; 2 Sanya Central Hospital (Hainan Third People’s Hospital), Sanya 572000, China

**Keywords:** nondilated left ventricular cardiomyopathy, cardiac magnetic resonance, CMR feature tracking, myocardial strain, prognosis

## Abstract

**Background**: To investigate the predictive value of cardiac magnetic resonance (CMR) feature parameters for major adverse cardiovascular events (MACEs) in patients with nondilated left ventricular cardiomyopathy and reduced left ventricular ejection fraction (NDLVC-rLVEF). **Methods**: This single-center retrospective study enrolled patients with NDLVC-rLVEF who underwent CMR between January 2015 and May 2025. MACEs included cardiovascular death, implantable cardioverter–defibrillator (ICD) discharge, and hospitalization due to heart failure or arrhythmia. Multivariable Cox regression analysis was used to identify independent risk factors for MACEs. **Results**: A total of 160 patients were included (mean age: 50.83 ± 15.81 years; 114 males, 46 females), with a median follow-up time of 53.00 months (IQR: 32.25–82.00). During this period, 41 patients (25.63%) experienced MACEs, including 10 cases of cardiovascular death, 1 case of ICD discharge, and 30 cases of rehospitalization due to heart failure or arrhythmia. Multivariable Cox regression analysis revealed that right ventricular ejection fraction (RVEF) and left ventricular global radial strain (LVGRS) were independent predictors of MACEs in patients with NDLVC-rLVEF. Kaplan–Meier analysis further demonstrated that patients with RVEF < 37% or LVGRS < 13% had a significantly higher incidence of MACEs (*p* < 0.05). **Conclusions**: Multimodal CMR parameters (RVEF and LVGRS) have significant predictive value for adverse prognosis in patients with NDLVC-rLVEF, facilitating early risk stratification and clinical intervention.

## 1. Introduction

In 2023, the European Society of Cardiology (ESC) Cardiomyopathy Management Working Group introduced for the first time the phenotypic concept of nondilated left ventricular cardiomyopathy (NDLVC). This phenotype is characterized by the presence of nonischemic scar tissue or fatty infiltration in an otherwise normal myocardium, which may or may not be accompanied by regional or global wall motion abnormalities or may solely present as isolated left ventricular hypokinesia without scarring [1]. Notably, recent evidence suggests that NDLVC may be associated with a higher prevalence of pathogenic arrhythmogenic gene variants compared to dilated cardiomyopathy, underscoring its potential arrhythmic risk and the need for early identification and risk stratification [2].

Cardiac magnetic resonance (CMR) imaging is widely regarded as the noninvasive gold standard for comprehensive myocardial assessment, offering precise evaluation of cardiac morphology, function, tissue characterization, and viability [3]. Within the expanding toolkit of CMR, feature tracking (CMR-FT) has emerged as a robust and reproducible technique for quantifying myocardial deformation without the need for additional scanning or contrast administration [4,5]. This technique quantitatively derives global longitudinal strain (GLS), global circumferential strain (GCS), and global radial strain (GRS) strain parameters by analyzing myocardial tissue displacement, providing an objective and reproducible means of evaluating myocardial mechanical function [6,7]. However, the prognostic value of cardiac magnetic resonance (CMR) structural, functional, and myocardial strain parameters in patients with NDLVC and reduced left ventricular ejection fraction (NDLVC-rLVEF) remains unclear and requires systematic investigation.

Therefore, this study retrospectively analyzed CMR imaging and clinical data from patients with NDLVC-rLVEF to systematically evaluate the predictive value of cardiac function and strain parameters for major adverse cardiovascular events (MACEs). The findings are expected to provide imaging-based evidence for the early identification of high-risk patients and personalized treatment strategies.

## 2. Materials and Methods

### 2.1. Study Population

This study included patients with NDLVC-rLVEF who underwent CMR imaging at Beijing Anzhen Hospital between January 2015 and May 2025. The following were the inclusion criteria: (1) meeting the diagnostic criteria for NDLVC, as defined by the 2023 ESC Guidelines for the Management of Cardiomyopathies [1]; (2) left ventricular ejection fraction (LVEF) <50%; (3) availability of complete CMR imaging data; (4) absence of left ventricular dilation, specifically defined as no elevated left ventricular end-diastolic volume index on CMR (≤96 mL/m^2^ for women and ≤105 mL/m^2^ for men) [8]. Exclusion criteria comprised: (1) a history of structural heart disease (e.g., congenital or valvular heart disease) or ischemic heart disease—the latter defined as >50% stenosis in a major epicardial coronary artery on CCTA or CAG, coronary-type (subendocardial or transmural) late gadolinium enhancement on CMR, or prior coronary revascularization—and (2) significant image artifacts that precluded reliable diagnostic evaluation. The endpoint was a composite of MACEs, including cardiovascular death, implantable cardioverter–defibrillator (ICD) discharge, and hospitalization due to heart failure or arrhythmia. Hospitalization for heart failure was defined as admission primarily due to signs and symptoms of heart failure. If multiple events occurred in a single patient, only the first event was counted. Overall survival was defined as the duration from the date of CMR imaging to the occurrence of an MACE or the last follow-up (censoring). Follow-up data were collected via regular clinic visits and telephonic interviews, with the final follow-up completed on 31 May 2025. This study complied with the tenets of the Declaration of Helsinki and was approved by the Ethics Committee of Beijing Anzhen Hospital, Capital Medical University (Registration No. KS2025104). As this was a retrospective study, the requirement for informed consent was waived.

### 2.2. CMR Examination

CMR examinations were performed on a 3.0 T scanner (Ingenia, Philips Healthcare, Best, The Netherlands) using a standardized protocol, equipped with a dedicated 32-channel cardiac phased-array surface coil, electrocardiographic gating, and respiratory gating techniques. Cine images were acquired using a steady-state free precession (SSFP) sequence, with imaging planes including the two-chamber, four-chamber, and short-axis views. The main scanning parameters were as follows: field of view, 286 mm × 340 mm; matrix, 180 × 200; repetition time, 3.4 ms; echo time, 1.7 ms; slice thickness, 8 mm; flip angle, 45°; and parallel imaging acceleration factor, 2.

### 2.3. CMR Data Analysis

#### 2.3.1. Measurement of Left and Right Ventricular Function

Left and right ventricular function parameters were analyzed using the CVI42 post-processing software 6.1.3 (Circle Cardiovascular Imaging Inc., Calgary, AB, Canada). The endocardial and epicardial contours of the left ventricle were semiautomatically delineated and manually corrected to obtain functional parameters, including ejection fraction (EF), end-diastolic volume (EDV), end-systolic volume (ESV), stroke volume (SV), cardiac output (CO), and left ventricular myocardial mass (LVMM). The ventricular outflow tract level was excluded from the analysis, with the basal slice operationally defined as the slice in which the left ventricular myocardium extended over ≥50% of its circumference; slices above this level containing the aortic valve or pure outflow tract were excluded. The papillary muscles were included in the ventricular blood pool. The ventricular cavities encompassed both papillary muscles and trabeculations. All images were analyzed by two attending radiologists, each with >5 years of experience in CMR diagnosis, using a double-blinded method for data measurement.

#### 2.3.2. Analysis of Left Ventricular Myocardial Strain

Left ventricular myocardial strain parameters were analyzed using the CVI42 software on SSFP cine sequences. The endocardial and epicardial contours of the left ventricle were semiautomatically traced and manually corrected to compute three-dimensional (3D) myocardial strain parameters. The 3D left ventricular myocardial strain parameters included GRS, GCS, and GLS.

### 2.4. Statistical Analysis

Statistical analysis was performed using SPSS software (version 26.0). Continuous variables were tested for normality. Those conforming to a normal distribution were expressed as mean ± standard deviation ($\bar{x}$ ± s) and compared between groups using the independent-sample t-test. Non-normally distributed data were presented as median (interquartile range) and compared using the Mann–Whitney U test. Categorical data were expressed as numbers (percentages) and compared using the chi-square test. The diagnostic performance of strain parameters in discriminating between the NDLVC and control groups was assessed using receiver operating characteristic (ROC) curve analysis.

## 3. Results

**Figure 1:**
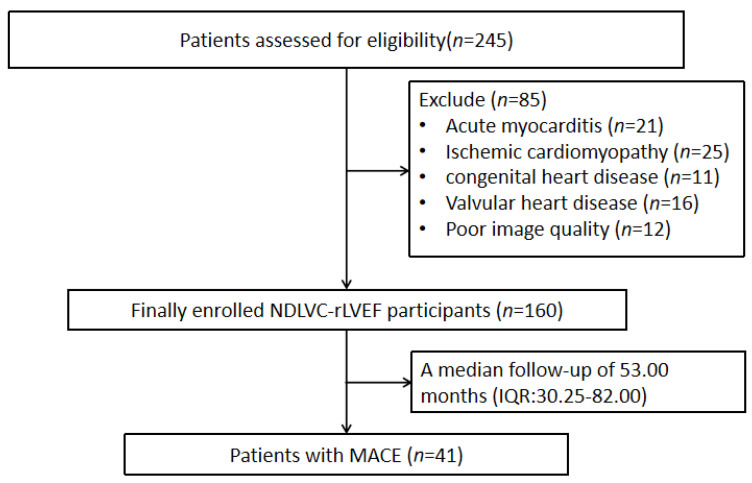
Flowchart of NDLVC patient inclusion. MACEs, major adverse cardiovascular events; NDLVC-rLVEF, nondilated left ventricular cardiomyopathy with reduced left ventricular ejection fraction; IQR, interquartile range.

A total of 245 patients initially met the inclusion criteria. After excluding 21 patients with acute myocarditis, 25 with ischemic cardiomyopathy, 11 with congenital heart disease, 16 with valvular heart disease, and 12 with poor image quality, 160 patients with NDLVC were ultimately included in this study. Based on the occurrence of MACEs, the patients were categorized into an event group (*n* = 41) and a non-event group (*n* = 119). During a median follow-up period of 53.00 months (interquartile range: 32.25–82.00), 41 patients experienced MACEs, including cardiovascular death (10 cases), ICD discharge (1 case), and rehospitalization due to heart failure or arrhythmia (30 cases). The flowchart of patient inclusion is illustrated in Figure 1.

### 3.1. Clinical Characteristics

Table 1 summarizes the baseline characteristics of all patients. In patients with NDLVC-rLVEF, there were no statistically significant differences between the MACE and non-MACE groups in terms of age, sex, height, weight, or body mass index. Patients with NDLVC who experienced MACEs had a higher heart rate than those without MACEs (*p* < 0.001). However, no statistically significant differences were observed between the MACE and non-MACE groups in the prevalence of hypertension, diabetes, hyperlipidemia, smoking, or alcohol consumption.

### 3.2. CMR Parameters of Left and Right Ventricular Function and Left Ventricular Myocardial Strain

Compared with the non-MACE group, patients with NDLVC-rLVEF who experienced MACEs showed significantly higher left ventricular end-systolic volume (LVESV, 127.83 [interquartile range, 94.68–150.41] vs. 97.89 [interquartile range, 79.54–118.38], *p* < 0.001) and right ventricular end-systolic volume (RVESV, 94.51 [interquartile range, 69.21–124.01] vs. 67.33 [interquartile range, 55.20–90.45], *p* < 0.001), whereas left ventricular stroke volume (LVSV, 46.77 [interquartile range, 32.39–56.89] vs. 60.36 [interquartile range, 45.09–71.10], *p* < 0.001), left ventricular cardiac output (LVCO, 3.65 [interquartile range, 2.68–4.17] vs. 4.24 [interquartile range, 3.26–5.24], *p* = 0.003), LVEF (28.62 [interquartile range, 20.23–32.81] vs. 36.78 [interquartile range, 31.45–42.02], *p* < 0.001), right ventricular stroke volume (RVSV, 38.67 [interquartile range, 26.01–48.47] vs. 55.10 [interquartile range, 38.90–67.26], *p* < 0.001), right ventricular cardiac output (RVCO, 2.91 [interquartile range, 2.10–4.02] vs. 3.82 [interquartile range, 2.92–4.73], *p* = 0.006), and right ventricular ejection fraction (RVEF, 29.43 ± 10.70 vs. 42.75 ± 10.84, *p* < 0.001) were significantly lower. No statistically significant differences were observed between the two groups in left ventricular end-diastolic volume (LVEDV), LVMM, or right ventricular end-diastolic volume (RVEDV).

**Figure 2:**
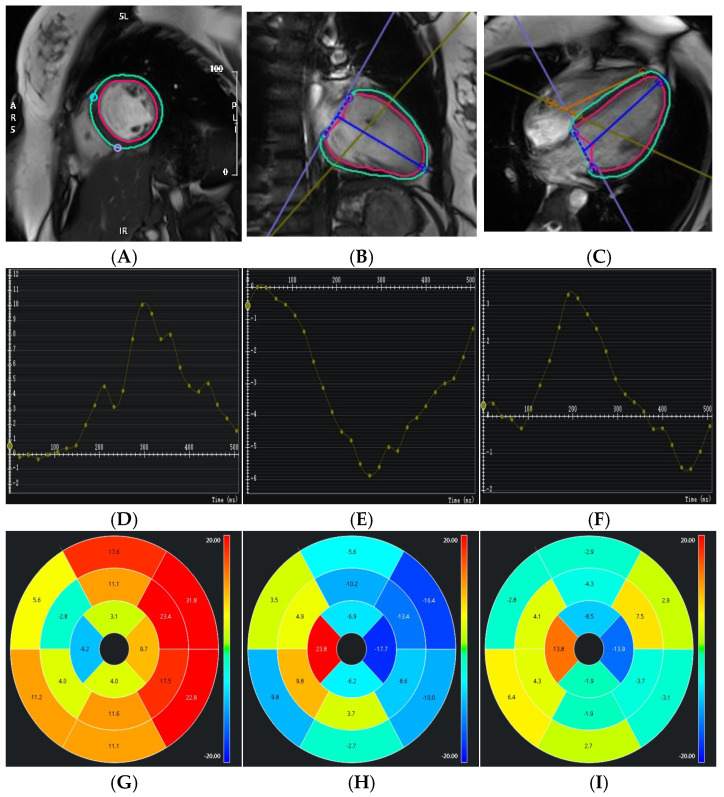
Example MRI studies of a 23-year-old man with NDLVC-rLVEF who was hospitalized for heart failure 8 months after the cardiac MRI examination. (**A**–**C**): Left ventricular strain images in the short-axis, two-chamber, and four-chamber views, respectively. The contours of the left ventricular endocardium (red) and epicardium (green) are delineated. (**D**–**F**): Strain curves for 3D left ventricular global radial strain (GRS), global circumferential strain (GCS), and global longitudinal strain (GLS), respectively. (**G**–**I**): Sixteen-segment bull’s-eye maps displaying the distribution of GRS, GCS, and GLS, respectively.

**Figure 3:**
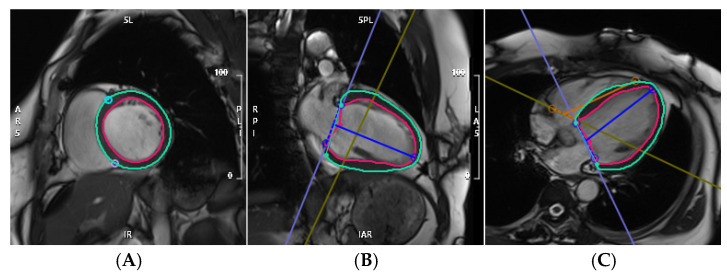

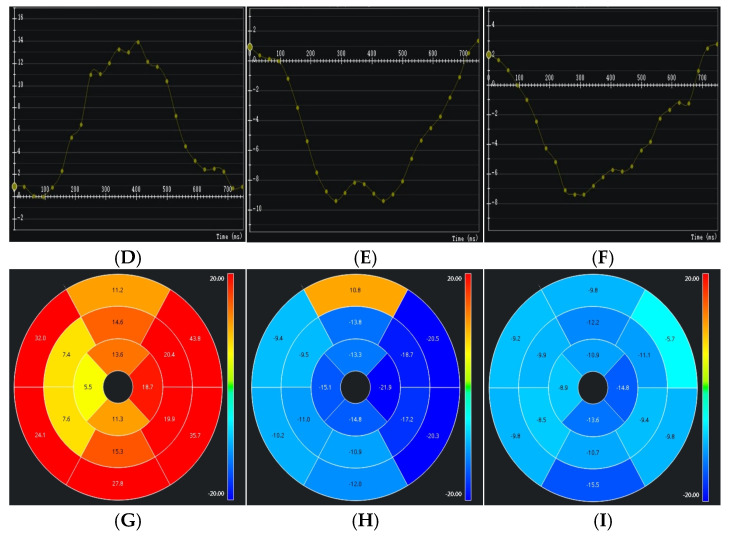
Example MRI studies of a 46-year-old man with NDLVC-rLVEF who had no adverse cardiovascular events at follow-up. (**A**–**C**): Left ventricular strain images in the short-axis, two-chamber, and four-chamber views, respectively. The contours of the left ventricular endocardium (red) and epicardium (green) are delineated. (**D**–**F**): Strain curves for 3D left ventricular global radial strain (GRS), global circumferential strain (GCS), and global longitudinal strain (GLS), respectively. (**G**–**I**): Sixteen-segment bull’s-eye maps displaying the distribution of GRS, GCS, and GLS, respectively.

Myocardial strain was impaired in patients with NDLVC-rLVEF who experienced MACEs, showing decreased LVGRS (10.53 [interquartile range, 6.60–15.27] vs. 18.09 [interquartile range, 14.78–22.21], *p* < 0.001), as well as reduced LVGCS (−8.30 [interquartile range, −11.26 to −5.13] vs. −12.37 [interquartile range, −15.04 to −10.41], *p* < 0.001) and LVGLS (−7.42 [interquartile range, −9.93 to −5.83] vs. −11.23 [interquartile range, −13.57 to −9.25], *p* < 0.001) (Table 2). Representative cases of patients with NDLVC with and without MACEs are shown in Figure 2 and Figure 3, respectively.

### 3.3. Association Between CMR/Clinical Risk Factors and Outcomes

**Figure 4:**
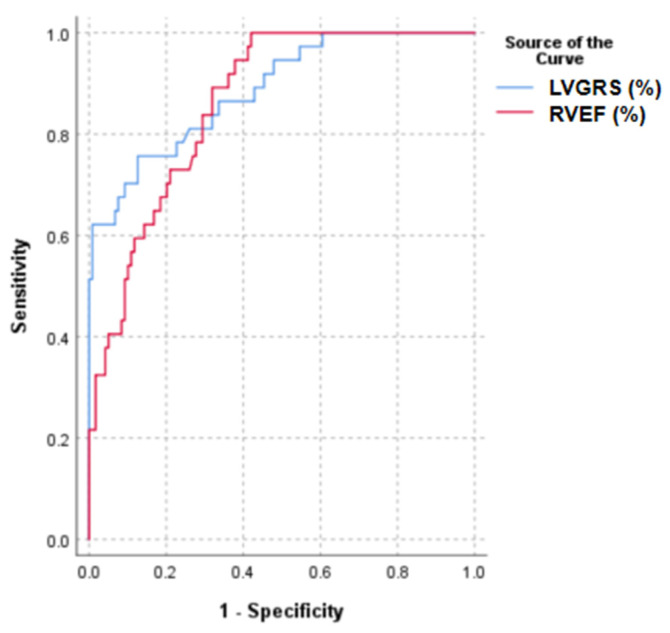
ROC curve. The ROC curve for the performance of CMR indexes for MACEs. RVEF = left ventricular ejection fraction; LVGRS = left ventricular global radial strain.

**Figure 5:**
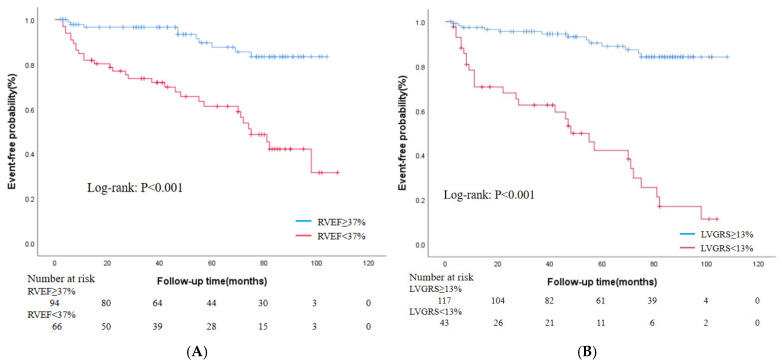
Kaplan–Meier survival curves. (**A**) Stratified by RVEF in all patients. (**B**) Stratified by GRS in all patients. RVEF = right ventricular ejection fraction; LVGRS = left ventricular global radial strain.

The results of univariate and multivariate Cox regression analyses are presented in Table 3. Univariate Cox proportional hazards model analysis revealed that LVSV, LVEF, RVSV, RVEF, LVGRS, and LVGLS were significantly associated with MACEs (*p* < 0.05). In the multivariate analysis, RVEF (hazard ratio [HR]: 0.953, 95% confidence interval [CI]: 0.911–0.997, *p* = 0.035) and LVGRS (HR: 0.859, 95% CI: 0.769–0.959, *p* = 0.007) remained independent factors significantly associated with MACEs. ROC analysis revealed that the optimal cutoff values for RVEF and LVGRS were 37% and 13%, respectively. The corresponding sensitivities were 80.50% and 68.30%, and specificities were 68.10% and 87.40%. The area under the curve values were 0.807 and 0.851, respectively (Figure 4). Kaplan–Meier curve analysis demonstrated that patients with NDLVC-rLVEF and RVEF < 37% or GRS < 13% had a significantly higher incidence of MACEs and markedly shorter survival times (*p* < 0.05) (Figure 5).

## 4. Discussion

This study evaluated the prognostic value of CMR-FT parameters in patients with NDLVC-rLVEF. The following were the major findings: (1) Of the patients with NDLVC-rLVEF, those who experienced MACEs showed significantly increased LVESV, decreased LVEF, and worse myocardial strain. (2) RVEF and LVGRS were significant independent predictors of adverse events in this patient population. (3) Patients with NDLVC with RVEF < 37% or LVGRS < 13% were significantly associated with a higher incidence of MACEs. These findings may help identify high-risk patients requiring close monitoring and follow-up, provide an objective imaging basis for early clinical identification and intervention, and contribute to precise phenotyping and individualized therapeutic strategies.

### 4.1. Characteristics of Patients with NDLVC-rLVEF

The introduction of the NDLVC phenotype in the 2023 ESC guidelines underscores the importance of phenotypic characterization, genetic testing, and CMR in the diagnosis and risk stratification of cardiomyopathies. This approach enables accurate identification and diagnosis of various cardiomyopathy subtypes, thereby supporting precise clinical management [9,10,11]. Several studies have investigated the role of CMR in phenotypic stratification and prognostic evaluation of NDLVC. Evidence suggests that CMR-based phenotyping can provide effective risk stratification in patients with NDLVC, with the extent of late gadolinium enhancement (LGE) serving as an objective imaging marker for identifying high-risk individuals. These findings support the integration of LGE assessment into routine clinical evaluation [12]. Other studies have compared the clinical profiles and outcomes of patients with NDLVC-rLVEF and those with dilated cardiomyopathy (DCM). Their results indicated no significant difference in the incidence of cardiac events between the two groups, highlighting the need for equally vigilant follow-up and regular assessment of left ventricular function in patients with both NDLVC and DCM [13]. This finding highlights the significance of establishing standardized follow-up protocols for patients with NDLVC.

CMR-FT utilizes high-resolution cine sequences to track the motion of the endocardial and epicardial borders throughout the cardiac cycle, enabling the precise quantification of myocardial tissue displacement and the calculation of GLS, GCS, and GRS [5,14,15]. This technique not only aids in the early diagnosis of cardiovascular diseases but also plays a critical role in the dynamic evaluation of disease progression and prognosis [16,17]. Previous studies have demonstrated the high diagnostic performance of CMR-FT in the diagnosis and differential diagnosis of various cardiovascular conditions [18,19,20]. Myocardial strain parameters have been reported to detect functional impairment earlier and more sensitively than conventional LVEF when myocardial injury occurs due to disease, thereby providing a valuable window for timely clinical intervention [21]. Tang HS et al. [22] observed that left ventricular GLS is a significant independent predictor of adverse outcomes in patients with DCM, offering substantial prognostic value.

In this study, compared to patients with NDLVC-rLVEF without MACEs, those who experienced MACEs exhibited altered biventricular function, characterized by significantly increased LVESV and RVESV; decreased LVSV, LVCO, LVEF, RVSV, RVCO, and RVEF; and impaired left ventricular strain, reflected by reduced absolute values of LVGRS, LVGCS and LVGLS. These findings indicate that patients with NDLVC-rLVEF suffer from impaired biventricular structure and function, accompanied by reduced CO and EF.

### 4.2. Prognostic Value of Biventricular Function and Left Ventricular Myocardial Strain Parameters in Patients with NDLVC-rLVEF

Multivariable Cox regression and survival analysis performed in this study identified RVEF and LVGRS as independent predictors of MACEs in patients with NDLVC-rLVEF. This finding suggests that alterations in biventricular structure and function, combined with impaired myocardial mechanics, collectively impact clinical outcomes.

Notably, strain parameters (GLS, GCS, and GRS) provide more sensitive information on myocardial mechanics than conventional EF, aiding in the detection of early myocardial dysfunction. By performing Kaplan–Meier analysis, this study further established cutoff values for these parameters: RVEF < 37% or GRS < 13%. Patients with values below these thresholds exhibited a significantly increased risk of MACEs and reduced survival, providing actionable criteria for risk stratification in clinical practice. CMR-FT technology is noninvasive, contrast-agent-free, and offers good reproducibility and feasibility, making it ideal for long-term follow-up and dynamic monitoring of patients with NDLVC, a considerably heterogeneous population.

### 4.3. Limitations

This study has several limitations. First, as a single-center retrospective study, our findings may be influenced by local clinical protocols, referral patterns, and patient-management approaches. Variations in care standards across institutions could affect the generalizability of the results, and validation in multicenter, prospective settings is warranted. Second, the current analysis only included left ventricular strain parameters and did not incorporate right ventricular or atrial strain data. Moreover, T1 mapping, extracellular volume fraction, late gadolinium enhancement (LGE) or genetic testing results were also not integrated. Hence, future studies should aim to incorporate multimodal parameters to develop a more comprehensive prognostic evaluation model.

## 5. Conclusions

This study demonstrates that multimodal cardiac magnetic resonance (CMR) parameters, specifically right ventricular ejection fraction (RVEF) and left ventricular global radial strain (LVGRS), serve as independent predictors of major adverse cardiovascular events (MACEs) in patients with non-dilated left ventricular cardiomyopathy and reduced ejection fraction (NDLVC-rLVEF). The findings indicate that impaired RVEF (<37%) and reduced LVGRS (<13%) identify a high-risk subgroup with significantly worse clinical outcomes. These results support the integration of these quantitative CMR markers into routine clinical assessment for early risk stratification, which may enhance prognostic accuracy and help guide personalized management in NDLVC-rLVEF patients.

## Figures and Tables

**Table 1:** Baseline characteristics of patients with NDLVC-rLVEF who reached and did not reach the endpoint.

Parameters	All Patients (*n* = 160)	Patients Who Did Not Reach the Endpoint (*n* = 119)	Patients Who Reached the Endpoint (*n* = 41)	*p* Value
Sex				0.092
Men	114 (71.25)	89 (74.79)	25 (61.00)	
Women	46 (28.75)	30 (25.21)	16 (39.00)	
Age (years) *	52.00 (38.25, 62.00)	53.00 (38.00, 62.00)	50.00 (37.50, 61.50)	0.302
Height (cm) *	168.00 (163.00, 172.75)	168.00 (164.00, 173.00)	167.00 (160.00, 171.50)	0.097
Weight (kg) *	68.00 (60.00, 76.00)	68.00 (60.00, 77.00)	67.00 (60.00, 75.00)	0.593
BMI (kg/m^2^) *	24.94 (22.65, 26.56)	24.98 (22.14, 26.53)	24.73 (23.05, 27.70)	0.632
Heart rate (bpm) *	72.50 (64.00, 82.70)	70.00 (64.00, 80.75)	77.00 (66.58, 90.25)	0.015
Hypertension, *n* (%)	60 (37.50)	42 (35.29)	18 (43.90)	0.326
Diabetes, *n* (%)	37 (23.13)	27 (22.69)	10 (24.39)	0.824
Hyperlipidemia, *n* (%)	32 (20.00)	25 (21.01)	7 (17.07)	0.587
Smoking, *n* (%)	53 (33.13)	37 (31.09)	16 (39.02)	0.352
Alcohol, *n* (%)	38 (23.75)	26 (21.85)	12 (29.27)	0.336

Note: Unless otherwise specified, data are numbers of patients, with percentages in parentheses. BMI = body mass index. * Data are medians, with interquartile ranges in parentheses.

**Table 2:** CMR parameter data of left and right ventricular cardiac function and left ventricular myocardial stress in patients with NDLVC-rLVEF who reached and did not reach the endpoint.

Parameters	All Patients (*n* = 160)	Patients Who Did Not Reach the Endpoint (*n* = 119)	Patients Who Reached the Endpoint (*n* = 41)	*p* Value
LVEDV (mL)	165.23 ± 44.05	162.71 ± 44.70	174.35 ± 43.25	0.149
LVESV (mL) *	103.61 (81.67, 132.40)	97.89 (79.54, 118.38)	127.83 (94.68, 150.41)	<0.001
LVSV (mL) *	55.43 (42.43, 67.60)	60.36 (45.09, 71.10)	46.77 (32.39, 56.89)	<0.001
LVCO (L·min^−1^) *	4.03 (3.05, 4.94)	4.24 (3.26, 5.24)	3.65 (2.68, 4.17)	0.003
LVEF (%) *	35.11 (28.93, 40.93)	36.78 (31.45, 42.02)	28.62 (20.23, 32.81)	<0.001
LVMM (g) *	104.90 (86.40, 123.10)	104.43 (84.25, 124.40)	109.62 (87.72, 122.22)	0.607
RVEDV (mL) *	126.59 (99.75, 155.75)	121.82 (98.75, 151.06)	131.43 (102.63, 172.89)	0.147
RVESV (mL) *	70.25 (56.82, 96.82)	67.33 (55.20, 90.45)	94.51 (69.21, 124.01)	<0.001
RVSV (mL) *	47.88 (36.13, 64.00)	55.10 (38.90, 67.26)	38.67 (26.01, 48.47)	<0.001
RVCO (L·min^−1^) *	3.62 (2.42, 4.61)	3.82 (2.92, 4.73)	2.91 (2.10, 4.02)	0.006
RVEF (%)	39.10 ± 12.31	42.75 ± 10.84	29.43 ± 10.70	<0.001
LVGRS (%) *	16.45 (12.88, 20.83)	18.09 (14.78, 22.21)	10.53 (6.60, 15.27)	<0.001
LVGCS (%) *	−11.62 (−13.94, −9.26)	−12.37 (−15.04, −10.41)	−8.30 (−11.26, −5.13)	<0.001
LVGLS (%) *	−10.67 (−12.70, −8.00)	−11.23 (−13.57, −9.25)	−7.42 (−9.93, −5.83)	<0.001

Note: Unless otherwise specified, data are means ± SDs. LVEDV = left ventricular end-diastolic volume; LVESV = left ventricular end-systolic volume; LVSV = left ventricular stroke volume; LVCO = left ventricular cardiac output; LVEF = left ventricular ejection fraction; LVMM = left ventricular myocardial mass; RVEDV = right ventricular end-diastolic volume; RVESV = right ventricular end-systolic volume; RVSV = left ventricular stroke volume; RVCO = right ventricular cardiac output; RVEF = right ventricular ejection fraction; LVGRS = left ventricular global radial strain; LVGCS = left ventricular global circumferential strain; LVGLS = left ventricular global longitudinal strain. * Data are medians, with interquartile ranges in parentheses.

**Table 3:** Univariate and multivariate Cox proportional hazards analysis of CMR measurements associated with major adverse cardiac events in patients with NDLVC-rLVEF.

Parameters	Univariable Analyses		Multivariable Analyses	
	Hazard Ratio	*p* Value	Hazard Ratio	*p* Value
Age (years)	0.991 (0.970, 1.013)	0.426		
BMI (kg/m^2^)	0.999 (0.917, 1.088)	0.977		
Heart rate (bpm)	1.002 (0.984, 1.020)	0.811		
LVSV (mL)	0.968 (0.951, 0.984)	<0.001	1.007 (0.975, 1.041)	0.663
LVEF (%)	0.900 (0.871, 0.930)	<0.001	0.978 (0.902, 1.061)	0.591
LVMM (g)	1.002 (0.992, 1.011)	0.720		
RVSV (mL)	0.964 (0.945, 0.983)	<0.001	1.007 (0.976, 1.038)	0.664
RVEF (%)	0.915 (0.888, 0.942)	<0.001	0.953 (0.911, 0.997)	0.035
LVGRS (%)	0.793 (0.742, 0.8247)	<0.001	0.859 (0.769, 0.959)	0.007
LVGLS (%)	1.119 (1.074, 1.167)	<0.001	1.008 (0.939, 1.081)	0.832

Note: Data in parentheses are 95% CIs. BMI = body mass index; LVSV = left ventricular stroke volume; LVEF = left ventricular ejection fraction; LVMM = left ventricular myocardial mass; RVSV = left ventricular stroke volume; RVEF = right ventricular ejection fraction; LVGRS = left ventricular global radial strain; LVGLS = left ventricular global longitudinal strain.

## Data Availability

The data that support the findings of this study are available from the corresponding authors upon reasonable request.

## Abbreviations

CMR = cardiac magnetic resonance; MACEs = major adverse cardiovascular events; NDLVC-rLVEF = nondilated left ventricular cardiomyopathy and reduced left ventricular ejection fraction; ICD = implantable cardioverter–defibrillator; NDLVC = nondilated left ventricular cardiomyopathy; CMR-FT = cardiac magnetic resonance feature tracking; GLS = global longitudinal strain; GCS = global circumferential strain; GRS = global radial strain; LVEF = left ventricular ejection fraction; SSFP = steady-state free precession; CVI = circle cardiovascular imaging; EF = ejection fraction; EDV = end-diastolic volume; ESV = end-systolic volume; SV = stroke volume; CO = cardiac output; LVMM = left ventricular myocardial mass; ROC = receiver operating characteristic.

## References

[1] Arbelo E., Protonotarios A., Gimeno J.R., Arbustini E., Barriales-Villa R., Basso C., Bezzina C.R., Biagini E., Blom N.A., De Boer R.A. (2023). 2023 ESC Guidelines for the management of cardiomyopathies. Eur. Heart J..

[2] Narducci M.L., Scacciavillani R., Nano R.L., Bisignani A., D’Alessandris N., Inzani F., Tiziano F.D., Perna F., Bencardino G., Burzotta F. (2024). Prognostic value of electroanatomic-guided endomyocardial biopsy in patients with myocarditis, arrhythmogenic cardiomyopathy and non dilated left ventricular cardiomyopathy. Int. J. Cardiol..

[3] Yang W., Xu J., Zhu L., Zhang Q., Wang Y., Zhao S., Lu M. (2023). Myocardial Strain Measurements Derived from MR Feature-Tracking: Influence of Sex, Age, Field Strength, and Vendor. JACC Cardiovasc Imaging.

[4] Hu M., Shen Y., Yu H., Song Y., Zheng T., Hong D., Gong L. (2023). Prognostic value of cardiac magnetic resonance imaging feature tracking technology in patients with light chain amyloidosis. Clin. Radiol..

[5] Zhang X., Wang C., Huang Y., Zhang S., Xu J. (2024). Unveiling the Diagnostic Value of Strain Parameters Across All 4 Cardiac Chambers in Patients with Acute Myocarditis with Varied Ejection Fraction: A Cardiovascular Magnetic Resonance Feature-Tracking Approach. J. Am. Hear. Assoc..

[6] Cai Q., Zhao Z., Gao J., Liu J., Li J., Peng X., Chen H. (2025). Normal Values for Atrial Deformation Measured by Feature-Tracking Cardiac MRI: A Meta-Analysis. J. Magn. Reson. Imaging.

[7] Shen M., Yang Z., Guo Y., Shi K., Jiang L., Wang J., Yan W., Qian W., Shen L., Li Y. (2025). Impact of Functional Mitral Regurgitation on Left Ventricular Strain in Nonischemic Dilated Cardiomyopathy Patients with Type 2 Mellitus Diabetes: A Magnetic Resonance Feature Tracking Study. J. Magn. Reson. Imaging.

[8] Castrichini M., De Luca A., De Angelis G., Neves R., Paldino A., Dal Ferro M., Barbati G., Medo K., Barison A., Grigoratos C. (2024). Magnetic Resonance Imaging Characterization and Clinical Outcomes of Dilated and Arrhythmogenic Left Ventricular Cardiomyopathies. J. Am. Coll. Cardiol..

[9] Miaris N. (2024). Non-dilated left ventricular non-compaction cardiomyopathy with systolic dysfunction is reclassified as non-dilated left ventricular cardiomyopathy with hypertrabeculation. Int. J. Cardiol..

[10] Filomena D., Vandenberk B., Dresselaers T., Willems R., Masci P.G., Robyns T., Bogaert J. (2025). Cardiac Diagnoses and Long-Term Outcomes in Ring-Like Late Gadolinium Enhancement Evaluated by Cardiac Magnetic Resonance. Eur. Hear. J.-Cardiovasc. Imaging.

[11] Kasiak P.S., Buchalska B., Kowalczyk W., Wyszomirski K., Krzowski B., Grabowski M., Balsam P. (2022). The Path of a Cardiac Patient-From the First Symptoms to Diagnosis to Treatment: Experiences from the Tertiary Care Center in Poland. J. Clin. Med..

[12] Jiang M., Zhou W., Qiao H.Y., Zheng T., Lian X., Wang Y., Yang W., Zhu L., Xu J., Zhou D. (2025). Phenotypic stratification and prognostic value of cardiac magnetic resonance in non-dilated left ventricular cardiomyopathy. Heart.

[13] Eda Y., Nabeta T., Iikura S., Takigami Y., Fujita T., Iida Y., Ikeda Y., Ishii S., Ako J. (2024). Non-dilated left ventricular cardiomyopathy vs. dilated cardiomyopathy: Clinical background and outcomes. ESC Hear. Fail..

[14] Koizumi S., Keiichi I., Sakai T., Kubota Y., Yokota H., Takaoka H., Kohno H., Matsumiya G. (2024). Cardiac Magnetic Resonance Feature Tracking Analysis for Change in Right Ventricular Function After Cardioplegic Arrest. Hear. Lung Circ..

[15] Matusik P.S., Mikrut K., Bryll A., Popiela T.J., Matusik P.T. (2025). Cardiac Magnetic Resonance Imaging in Diagnostics and Cardiovascular Risk Assessment. Diagnostics.

[16] Stojanovska J., Nijveldt R., Ordovas K., Vliegenthart R., Seiberlich N., Prieto C., Ojha V., Hanneman K., Lawton B., Hughes M. (2025). Highlights of the Cardiovascular Magnetic Resonance 2024 Conference: The first joint European Association of Cardiovascular Imaging, European Society of Cardiovascular Radiology, and Society for Cardiovascular Magnetic Resonance conference. J. Cardiovasc. Magn. Reson..

[17] Prieto C., Allen B.D., Azevedo C.F., Lima B.B., Lam C.Z., Mills R., Huisman M., Gonzales R.A., Weingärtner S., Christodoulou A.G. (2025). Highlights of the Society for Cardiovascular Magnetic Resonance 2025 conference: Leading the way to accessible, efficient, and sustainable cardiovascular magnetic resonance. J. Cardiovasc. Magn. Reson..

[18] Zhao Y., Song Y., Mu X. (2024). Application of left atrial strain derived from cardiac magnetic resonance feature tracking to predict cardiovascular disease: A comprehensive review. Heliyon.

[19] Santoro F., Vitale E., Ragnatela I., Cetera R., Leopzzi A., Mallardi A., Matera A., Mele M., Correale M., Brunetti N.D. (2024). Multidisciplinary approach in cardiomyopathies: From genetics to advanced imaging. Hear. Fail. Rev..

[20] Feher A., Del Galdo F., Plein S. (2024). Advances in the diagnosis of multiorgan involvement in systemic sclerosis: A focus on MRI. Curr. Opin. Rheumatol..

[21] Cionca C., Zlibut A., Agoston R., Agoston-Coldea L., Orzan R.I., Mocan T. (2024). Evaluating the Clinical Utility of Left Ventricular Strains in Severe AS: A Pilot Study with Feature-Tracking Cardiac Magnetic Resonance. Biomedicines.

[22] Tang H.S., Kwan C.T., He J., Ng P.P., Hai S.H.J., Kwok F.Y.J., Sze H.F., So M.H., Lo H.Y., Fong H.T.A. (2023). Prognostic Utility of Cardiac MRI Myocardial Strain Parameters in Patients with Ischemic and Nonischemic Dilated Cardiomyopathy: A Multicenter Study. Am. J. Roentgenol..

